# Imagerie de la cellulite orbitaire chez l’enfant: à propos de 56 cas

**DOI:** 10.11604/pamj.2018.30.94.14672

**Published:** 2018-05-31

**Authors:** Dounia Basraoui, Ayoub Elhajjami, Hicham Jalal

**Affiliations:** 1 Radiology Departement, Mother and Child’s Hospital, Mohammed VI^th^ Teaching Center, Medical School of Marrakesh, Cadi Ayad University, Marrakesh, Morocco

**Keywords:** Cellulite, orbitaires, TDM, CT, enfant, Cellulitis, orbit, CT scan, children

## Abstract

Les cellulites orbitaires de l'enfant sont rares mais potentiellement graves, le diagnostic est principalement basé sur l'examen clinique et l'imagerie (tomodensitométrie ou IRM). Le but de ce travail est d'illustrer le rôle de l'imagerie, essentiellement la TDM, dans le diagnostic et la prise en charge de cette pathologie. Il s'agit d'une étude rétrospective portant sur 56 cas de cellulite orbitaire, colligés durant six années au service de radiologie de l'hôpital mère-enfant du CHU Mohammed VI de Marrakech (de janvier 2011 à octobre 2017), explorés par TDM crânio-orbitaire. L'âge moyen des patients était de 5 ans, avec une légère prédominance féminine. La porte d'entrée était dominée par l'atteinte sinusienne retrouvée chez 22malades. L'analyse des résultats tomodensitométriques a noté 37 cas de cellulite préseptale (66%), 3 cas de cellulite rétroseptale isolée (5%), 16 cas de cellulite mixte (28%), 8 cas d'exophtalmie (14%) et 4 cas d'abcès sous-périosté (7%). Les cellulites orbitaires de l'enfant sont des infections graves, entravant le pronostic vital à court terme et le pronostic fonctionnel visuel à moyen et long terme, le diagnostic positif est clinique. L'imagerie joue un rôle important dans le diagnostic topographique et étiologique, ainsi que pour guider le traitement.

## Abstract (en)

Orbital cellulitis is rare but potentially severe in children. Diagnosis is primarily based on clinical examination and imaging (CT or MRI). This study aims to highlight the role of imaging, in particular CT scan, in the diagnosis and in the treatment of this pathology. We conducted a retrospective study of 56 cases of orbital cellulitis, whose data were collected in the Department of Radiology at the Hospital mother and child CHU Mohamed VI, Marrakech over a period of six years (January 2011-October 2017). Patients underwent cranio-orbital CT scan. The average age of patients was 5 years, with a slight female predominance. The disease mainly involved the sinus (22 patients). The analysis of computed tomography results highlighted 37 cases of preseptal cellulitis (66%), 3 cases of isolated retroseptal cellulitis (5%), 16 cases of mixed cellulitis (28%), 8 cases of exophthalmia (14%) and 4 cases of subperiosteal abscess (7%). Orbital cellulitis is a serious infection in children, affecting short term vital prognosis as well as medium-term and long-term functional visual prognosis. Positive diagnosis is based on clinical examination. Imaging plays an important role in topographical and etiological diagnosis and as guidance for treatment.

## Introduction

Les cellulites orbitaires de l'enfant sont rares mais potentiellement graves, leur diagnostic est basé principalement sur l'examen clinique et l'imagerie (tomodensitométrie ou IRM). La prise en charge thérapeutique urgente est basée sur une antibiothérapie parentérale parfois associée à un drainage chirurgical. Le but de notre travail est d'illustrer le rôle de l'imagerie, essentiellement la TDM, dans le diagnostic et la prise en charge des différentes lésions observées au cours d'une cellulite orbitaire.

## Méthodes

Il s'agit d'une étude rétrospective portant sur 56 cas de cellulite orbitaire colligés durant six années au service de radiologie de l'hôpital mère-enfant au CHU Mohammed VI de Marrakech (de janvier 2011 à octobre 2017), explorés par TDM crânio-orbitaire. Tous nos patients ont bénéficié d'une TDM cranio-orbitaire en urgence, avec des reconstructions dans les trois plans de l'espace et injection de produit de contraste iodé. Dans notre étude, nous avons adopté la classification de Chandler pour stadifier les lésions orbitaires.

## Résultats

**Figure 1:**
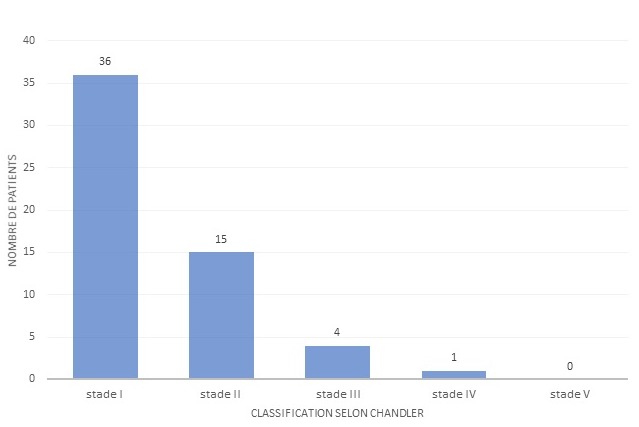
Répartition des cellulites orbitaires selon la classification de Chandler

**Figure 2:**
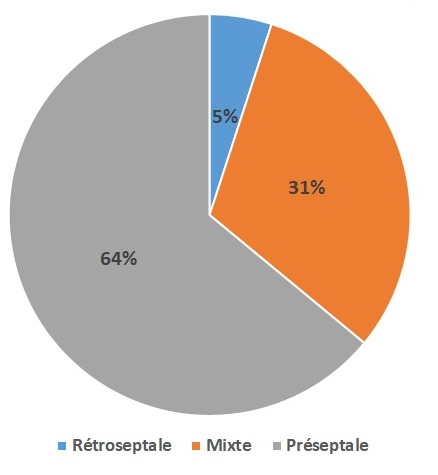
Répartition des cellulites orbitaires selon la topographie

Sur les 56 cas de cellulite faciale colligés, 29 (51,7%) étaient de sexe féminin et 27 (48,3%) de sexe masculin,soit un sex-ratio de 1,07. L'âge de nos patients variait entre 6 mois et 14 ans avec une moyenne de 5 ans.Les patients étaient d'origine urbaine dans 71,5% des cas, d'origine rurale dans 18% descas et péri-urbaine dans 10,5% des cas.Trente deux pour cent des malades étaient admis en période estivale (du mois de Juin à Aout), 25% des cas en période d'Automne (de Septembre à Décembre) et 21,5% des malades en périodesd'hiver et printemps chacun. Le signe fonctionnel principal ayant motivé la consultation était la tuméfactionpalpébrale, qui était constante chez tous les malades (100%). Elle était bilatérale dans 25% descas, les autres signes cliniques comportaient une exophtalmie chez 21,4% des malades, lechémosis, ptosis et la diminution de la mobilité oculaire dans 3,5% des cas chacun. Trente-sixmalades étaient fébriles soit 64% des cas. Tous les malades avaient un état général conservé et unexamen neurologique normal (pas de syndrome méningé, pas de déficit neurologique). La cellulite orbitaire dans notre étude était bilatérale chez 6 cas soit 10.7%, unilatérale pour 50 cas soit 89.3 % (22 du côté gauche et 28 du côté droit). la (Figure 1) représente la distribution des cellulites selon la classification de Chandler [1]. La topographie de la cellulite était préseptale chez 36 patients soit 64,28% notant l'atteinte des paupières, des canthus internes et externes et des glandes lacrymales; rétroseptale isolée chez 3 patients soit 5,3% avec l'atteinte des graisses intra et extra-coniques et parfois l'infiltration des muscles oculomoteurs et mixte pré et rétroseptale chez 17 patients soit 30,35%. La (Figure 2) représente la répartition des cellulites orbitaires selon la topographie. L'exophtalmie a été retrouvée chez seulement 8 patients (14,2%), au stade 1 chez 4 patients (7,1%) et au stade 2 chez 4 patients (7,1%). L'atteinte osseuse a été retrouvé chez 4 patients (7%) qui ont présenté des abcès sous-périostés et un aspect mité de la lame papyracée de l'éthmoïde. Aucun cas de thrombose du sinus caverneux n'a été retrouvé dans notre série. L'étiologie infectieuse dans notre série était prédominante, retrouvée chez 33 patients soit 58,9% des cas, parmi-elles la cause sinusienne arrive en chef de file chez 22 enfants (39,2% des cas), avec surtout l'ethmoïdite, suivi des pansinusites et quelques cas de sinusites fronto-maxillaires. Des portes d'entrée dentaire chez un seul patient, ophtalmique avec infiltration de la glande lacrymale et dacryolithiase chez 4 patients, cutanée chez 4enfants et un cas de cellulite sur prothèse oculaire ont été retrouvées. La cellulite orbitaire post-traumatique a été retrouvée chez 5 patients soit 9% des cas, l'étiologie est restée indéterminée chez 18 patients soit 32.14% des cas.

## Discussion

**Figure 3:**
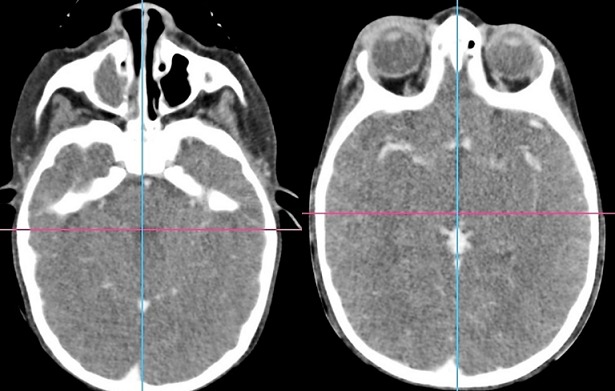
TDM, coupes axiales montrant unecellulite orbitaire droite rétro septale sans collection décelable d’origine sinusienne (Chandler 2)

**Figure 4:**
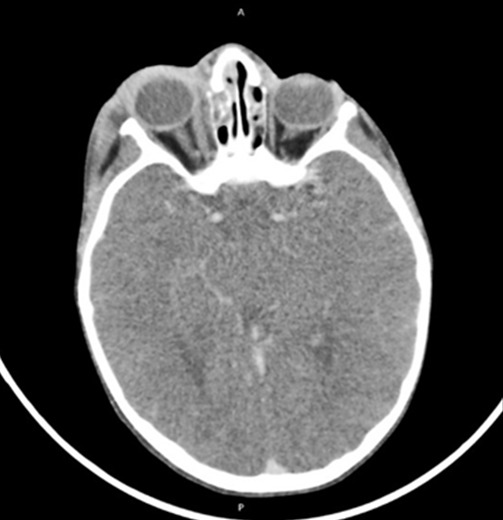
TDM, coupe axiale avec montrant une cellulite droite mixte (Chandler 2)

**Figure 5:**
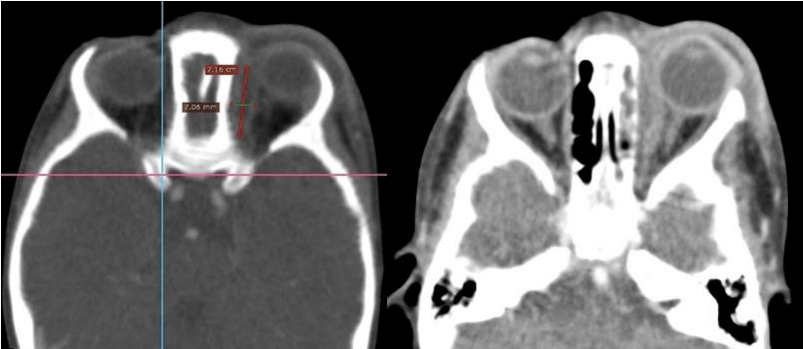
TDM, coupes axiales montrant une cellulite rétro septale compliquée d’abcès sous-périosté avec exophtalmie grade I, d’origine sinusienne (Chandler 3)

**Figure 6:**
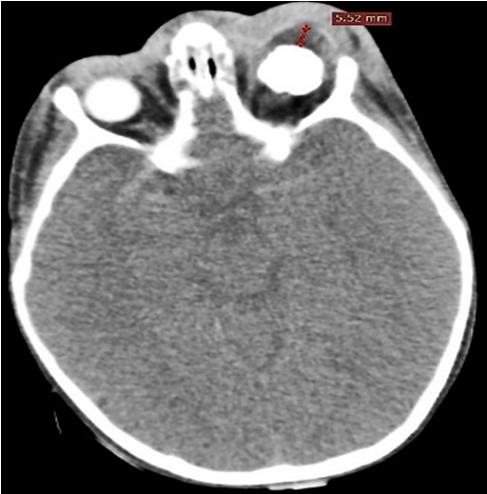
TDM, coupe axiale montrant une cellulite orbitaire gauche sur prothèse (Chandler 2)

**Figure 7:**
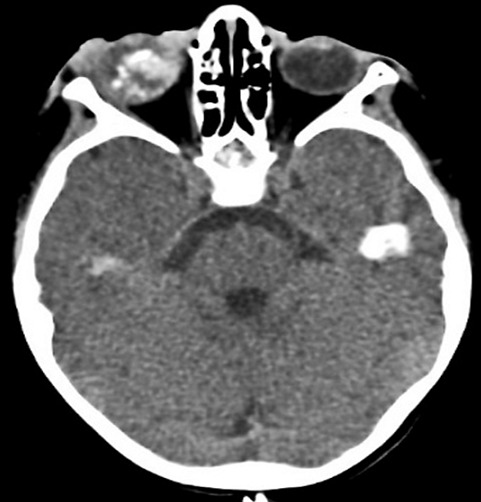
répartition des patients ayant une cellulite orbitaire selon l’étiologie

La cellulite orbitaire représente une cause relativement fréquente d'inflammationorbitaire. Chez l'enfant, elle serait responsable de 0,9% d'admissions en pédiatrie par anselon une série canadienne [2]. L'âge moyen des enfants ayant une cellulite orbitaire est variable entre 3 à 7 ans dans la majorité des séries pédiatriques publiées [2-4], ce qui concorde avec le résultat de notre étude qui était de 5 ans. Cliniquement la cellulite réalise un œdème inflammatoire de lœil, le plus souventunilatéral, douloureux et fébrile d'installation aiguë et de progression rapide [5]. L'imagerie est indispensable en cas de suspicion de celluliteorbitaire. La radiographie standard n'est plus indiquée. Unetomodensitométrieorbitaire avec et sans injection de produit de contraste est l'examen clé du diagnostic positif etparfoisétiologique, mais ne permet pas toujours de différencierabcès, œdème inflammatoire et hématome [6]. Enrevanche, la tomodensitométrie permettra de déterminer lalocalisationexacte, la taille de la lésion orbitaire et l'étatdessinus de la face [7]. En 1937, Hubert publie une classification des complications de la sinusitequi comprenait des complications palpébrales, orbitaires et intracrâniennes [8]. En 1970, Chandler a utilisé la classification de Hubert, pour décrire les complications orbitaires des sinusites [1]. Cette classification est aussi une gradation de l'évolutionde da cellulite. Ainsi, une cellulite pré-septale (grade I) peutévolueren l'absence de traitement vers un grade IV voireV. La cellulite pré-septale (grade I) qui est caractérisée par un œdème inflammatoire des paupières [6] et dont l'image typique est une hypodensité infiltrant la région pré-septale et augmentant le volume tissulaire constitue la forme clinique la plus fréquente, comme ce qui est rapporté dans la majorité des séries (73% à 93% des cas) [2, 4, 9, 10], dans notre série, la forme pré-septale concerne seulement 64% des cas ce qui peut être est expliqué par le retard de diagnostic dans notre contexte. Dans la cellulite de grade II, on retrouve un œdème palpébral important et diffus (Figure 3), qui est représenté par une hypodensité infiltrant les tissus, avec une graisse orbitaire hétérogène, d'aspect « sale » ou « moucheté », avec quelquefois la présence de phlegmon solide dans l'orbite médiale et un aspect de gros muscles [11] rehaussés après injection de produit de contraste, 15 cas ont été retrouvés dans notre étude soit 26% (Figure 4). L'abcès sous périosté (grade III) forme une densité confluente dans l'orbite médiale plus fréquemment, ou supérieure, avec une opacification du sinus ethmoïdal, un niveau hydro-aérique ou un aspect d'anneau péri-lésionnel se rehaussant après injection est pathognomonique [12], 4 cas ont été retrouvés dans notre étude soit 7% (Figure 5). L'Abcès orbitaire (grade IV) forme une collection de pus dans l'orbite avec infiltration diffuse de la graisse intra et extra-orbitaire [11], un seul cas a été retrouvé dans notre étude. La thrombophlébite de sinus caverneux (grade V) se traduit si elle est récente par une hyperdensité spontanée de la loge caverneuse. En cas de thrombophlébite ancienne, la TDM après injection de produit de contraste objective un défect endo-luminal du sinus caverneux [11]. En terme de fréquence, elle reste une complication rare [13]; aucun cas n'a été retrouvé dans notre série. Les cellulites orbitaires, bien que majoritairement secondairesà des sinusites, peuvent révéler diversesétiologies. Autravers de ce travail, nous avons tenté demontrer que chaque tableau d'inflammation orbitaire chezunenfant ne doit pas être étiqueté comme éthmoïdite sansunedémarche diagnostique soigneuse. Ladémarche diagnostique permet de ne pas méconnaîtredes étiologies plus inhabituelles, telles que les infections desvoies lacrymales (4 cas retrouvés dans notre étude), un corps étranger intra-orbitaire(prothèse oculaire (Figure 6), ou encore une tumeur telle qu'un rhabdomyosarcome ou un rétinoblastome (Figure 7). Lebilan étiologique passera obligatoirement par unexamenclinique soigneuxpuis une imagerie orbitaire [7], en urgence si nécessaire. Il s'agira en première intention d'une tomodensitométrie orbitaire [6] qui permettra de déterminer la localisation exacte des lésions et d'explorer les cavités sinusiennes. On recherchera un corps étranger intra-orbitaire, une masse tumorale et on s'attachera à préciser le retentissement sur le globe par la gradation de l'exophtalmie.

## Conclusion

La cellulite orbitaire chez l'enfant pose des difficultésdiagnostiques et thérapeutiques liées au polymorphismeétiologique. Face à un tableau clinique atypique, un bilan étiologique rapide est essentiel. Hormis lesétiologies sinusiennes, les infections des voies lacrymales etlestumeurs sont les principales causes à rechercher devant une cellulite orbitaire de l'enfant. Heureusement l'accèsdeplus en plus facile à une imagerie de l'orbite permet une orientation rapide et efficace.

### Etat des connaissances actuelles sur le sujet

- Les infections orbitaires de l'enfant ne posent le plus souvent pas de difficulté diagnostique devant des signes cliniques bruyants et d'aggravation rapide;
- La porte d'entrée des cellulites orbitaires est le plus souvent sinusienne.

### Contribution de notre étude à la connaissance

- Chaque tableau d'inflammation orbitaire chez un enfant ne doit pas être étiqueté comme éthmoïdite sans une démarche diagnostique soigneuse, basée sur l'imagerie.

## Conflits d’intérêts

Les auteurs ne déclarent aucun conflit d'intérêts.
